# Prevalence of active HCV infection among the blood donors of Khyber Pakhtunkwa and FATA region of Pakistan and evaluation of the screening tests for anti-HCV

**DOI:** 10.1186/1743-422X-8-154

**Published:** 2011-04-01

**Authors:** Najib U Khan, Ijaz Ali, Naeem U Ahmad, Aqib Iqbal, Latif U Rehman, Iqbal Munir, Muti U Rehman, Sanaullah Khan, Sajid Ali, Lubna Siddique, Zahoor A Swati

**Affiliations:** 1 Institute of Biotechnology and Genetic Engineering, KPK Agricultural University, Peshawar, Pakistan; 2 University of Veterinary and Animal Sciences, Lahore, Pakistan; 3 Kohat University of Science and Technology, Kohat, Pakistan; 4 Centre for Biotechnology, University of Pesawar, Pakistan; 5 Biodiversity Center, Islamia College University Peshawar, Pakistan

**Keywords:** anti HCV, immunochromatography, ELISA, RT-PCR, Pakistan

## Abstract

Hepatitis C is a fatal liver disease caused by the hepatitis C virus. In this study, blood donors, from various districts of the KPK province and the federally administered tribal area (FATA) of Pakistan were tested for anti-HCV antibodies and HCV RNA by ICT (Immuno-chromatographic test), ELISA and RT-PCR. Out of the 7148 blood donors, 224 (3.13%) were positive for anti-HCV antibodies by ICT, 135 (1.89%) by ELISA while 118 (1.65%) blood donors had active HCV infection as detected by RT-PCR. We suggest that ELISA should be used for anti-HCV screening in public sector hospitals and health care units.

## 1. Background

Hepatitis C is caused by the hepatitis C virus (HCV), which was identified in 1989 [1]. Hepatitis C virus has a positive sense single-stranded RNA genome. The genome consists of a single open reading frame that is 9600 nucleotide bases long [2]. Contaminated blood, blood products and body fluids are common modes of transmission of HCV. Other risk factors include intravenous drug abuse, use of barber razor, dental procedures, tattooing, ear piercing, acupuncture and high-risk sexual behavior [3]. About 3% of the world population is infected with hepatitis-C virus [4]. Laboratory diagnosis of HCV infection is usually made on the basis of the detection of circulating antibodies. Serological tests for detecting antibodies to HCV are generally classified as screening tests or confirmatory tests. The most widely used screening tests are ELISAs. Recently, other screening tests including agglutination, immunofilteration and immunochromatographic tests have been developed [5].

Earlier studies have reported the prevalence of anti-HCV antibodies among the blood donors or general population from KPK province (Previously called N-W.F.P) using Immunochromatographic tests [6-8] while active infection has never been investigated. With the purpose of investigating the prevalence of active HCV infection and analyzing the scope of antibody-based HCV detection for screening blood and blood products, we tested 7148 blood donors for anti-HCV or HCV RNA by ICT, ELISA and Real-time PCR. Our results indicated that the prevalence of anti-HCV antibodies as detected by ELISA as well as the prevalence of active HCV infection were lower as compared to the previous studies which were based on antibody-based tests alone while the prevalence of anti-HCV antibodies based on Immunochromatographic tests fell in the range of previously documented prevalence rates in KPK.

## 2. Methods

The aim of this study was to analyze the prevalence of the anti-HCV antibodies or HCV RNA among the blood donors of KPK and the federally administered areas (FATA) of Pakistan from January to September 2009. Three different methods were used to find out the prevalence of anti-HCV antibodies or HCV RNA. The scope of ICT and ELISA techniques for screening blood was also evaluated.

### Blood donors

Blood samples was taken from the voluntary blood donors and examined either at Hayat Abad Medical Complex (HMC) or at the Institute of Biotechnology and Genetic Engineering, KPK Agricultural University Peshawar.

### Immuno-chromatographic tests (ICT)

Initially all the blood donors were tested for anti-HCV antibodies by immuno-chromatographic tests. Each positive sample was tested twice. The immune-chromatographic strips used in this study were from two different sources. Samples positive by ICT technique were further evaluated using ELISA.

### ELISA

Sera positive by ICT were tested for anti-HCV antibodies by ELISA (BIOKIT, S.A, Barcelona-Spain) according to the manufacturer's instructions. All the ELISA positive samples were processed for RNA extraction.

### RNA Isolation and Real Time PCR

RNA isolation from the HCV positive ELISA samples and subsequent RT-PCR was carried out with the help of RNA extraction and RT-PCR kit from Sacace (Sacace, Biotechnology, Italy) according to the manufacturer's instructions, inside the Cepheid smart cycler (Nasdaq: CPHD, California, US).

## 3. Results

### HCV prevalence among the blood donors in KPK and FATA

A total of 7148 voluntary blood donors were initially screened for anti HCV antibody by ICT. 3.13% of the volunteers were detected positive for anti-HCV antibodies (Table 1). All the samples positive by ICT were further processed by ELISA which indicated that out of the total number of volunteers, 1.89% were positive for anti-HCV antibodies by ELISA (Table 1).

**Table 1:** Prevalence of anti HCV and HCV RNA among the blood donors of KPK and FATA region of Pakistan

MONTH	DONORS	Anti HCV (ICT^+^)	Anti HCV (ELISA^+^)	Real-time PCR^+ ^cases
JANUARY	974	34	21	20

FEBRUARY	1013	44	13	12

MARCH	972	23	15	14

APRIL	938	22	14	13

MAY	936	23	21	20

JUN	1095	25	18	18

JULY	673	23	12	11

AUGUST	547	30	11	10

8	**7148**	**224 (3.13%)**	**135 (1.89%)**	**118(1.65%)**

Samples positive by either ICT or ELISA were used for HCV RNA extraction and subsequent RT-PCR. The real-time PCR assay revealed that 118 (1.65%) donors had HCV RNA in their blood (Table 1).

## 4. Discussion

Viral hepatitis is rapidly spreading among the general population of Pakistan. The lack of proper screening facilities or expertise in screening blood and blood products for possible HCV infection at our public sector hospitals is partly contributing towards the spread of HCV. Previous studies on HCV prevalence based on anti-HCV antibodies among the blood donors have revealed high prevalence rate in KPK province [6,8-10]. High prevalence of HCV among the blood donors has also been reported from other parts of the country as well [11-14]. Majority of the studies undertaken have relied on the detection of antibodies against HCV and active infection among the blood donors has never been figured out. In our study, we have coupled the antibodies-based tests with modern RT-PCR based HCV RNA detection in order to exactly figure out the prevalence of active HCV infection among the blood donors from KPK and FATA region. Other studies from KPK mentioned earlier in this section have reported anti-HCV prevalence from 3 to 4%. In this study, screening of blood by ICT devices revealed that 3.13% of the blood donors were positive for anti-HCV which is well in the range of previously reported anti-HCV prevalence. As false positivity is a common problem associated with ICT devices [15-17] so in order to refine the screening procedure we analyzed all the ICT positive samples by 3^rd ^generation ELISA which indicated that 1.89% of the blood donors had antibodies against HCV. Seventeen (12.6%) blood donors who were positive for anti-HCV by ICT turned out to be negative by ELISA. These results reveal that screening of blood and blood products by ICT devices may not predict the true picture of anti-HCV prevalence. In KPK, screening of the blood and blood products at maximum health care units is carried out with ICT devices only. We suggest that ELISA should replace ICT procedures for screening in all health care units, especially those which are concerned with immigration of the workers. Companies are developing ELISAs for the detection of HCV core antigen. These assays can be used in addition to anti-HCV assays and may provide a valuable tool in the identification of individuals undergoing HCV seroconversion. These assays may be more appropriate as a supplement to anti-HCV antibody tests than using NAT for screening blood donations in countries with limited resources [5].

For confirmation of the active infection, we investigated all the ELISA positive samples by Real-time PCR which revealed that 1.65% of the blood donors had HCV RNA in their blood. Presence of anti-HCV and the absence of HCV RNA in the blood may be attributed to the self limiting nature of the disease in some people or it may be due to the presence of antibodies against HCV in treated subjects.

## Conclusion

Prevalence of active HCV infection among the blood donors of KPK province of Pakistan and the federally administered tribal region of Pakistan is 1.65% which is lower in comparison to the previous estimates. The scope of ICT devices for screening against anti-HCV seems to be limited. Proper and reliable HCV screening should include latest ELISA procedures.

## Competing interests

The authors declare that they have no competing interests.

## Authors' contributions

IA designed the study, advised about the protocols and prepared the manuscript. NK, LR and LS carried out ICT, ELISA and RT-PCR. MR, SK and SA helped in sampling. NA, AI, IM and ZAS critically reviewed the manuscript. All authors read and approved the final manuscript.
